# An accessory right hepatic artery derived from the superior mesenteric artery for anterior right liver lobe supply: a case report

**DOI:** 10.1007/s00276-018-2173-3

**Published:** 2018-12-22

**Authors:** Xiaowen Li, Xinjing Zhang, Qian Lu, Ang Li, Jingyi Lin, Haining Fan, Rui Tang

**Affiliations:** 1 Department of Hepatopancreatobiliary Surgery and Liver Transplantation Center, Tsinghua University Affiliated Beijing Tsinghua Changgung Hospital, No. 168 Litang Road, Changping District, Beijing, 102218 China; 2 Department of Hepatopancreatobiliary Surgery, Affiliated Hospital of Qinghai University, Xining, 810001 China

**Keywords:** Accessory right hepatic artery, Anatomical variation, Liver, Right anterior sector

## Abstract

**Purpose:**

During the last decades, it has been established that there are numerous individual anatomical variations of the arterial blood supply in human liver. In the present study, we examined the liver vascularization of an intrahepatic cholangiocarcinoma patient.

**Methods:**

For surgical planning, an enhanced CT scan was performed and a three-dimensional model of liver vascularization constructed.

**Results:**

The patient was diagnosed as a Michel’s type VII hepatic artery variation. An accessory right hepatic artery arose from the superior mesenteric artery and had distributed into the right anterior liver to provide the blood supply of segments V and VIII, which was more medial than the territory of the right hepatic artery coming from the proper hepatic artery. At the same time, an accessory left hepatic artery originated from the left gastric artery.

**Conclusion:**

We present a case in which an accessory right hepatic artery provided a territory more medial than a right hepatic artery coming from the proper right artery.

## Introduction

An accessory right hepatic artery (aRHA) refers to an additional artery of the right liver, while a normal branch derives from a proper hepatic artery (PHA) [3]. In general, aRHA arises mainly from the superior mesenteric artery (SMA), but may also branch off from the celiac trunk (CT), common hepatic artery (CHA), renal artery (RA), gastroduodenal artery (GDA), abdominal aorta (AA), and others. aRHA arising from the SMA is usually located at the right posterior side of the pancreas head, duodenum, and portal vein (PV). It runs to and provides an arterial blood supply to the right posterior liver (RPL) through the hepatoduodenal ligament.

## Case report

**Fig. 1:**
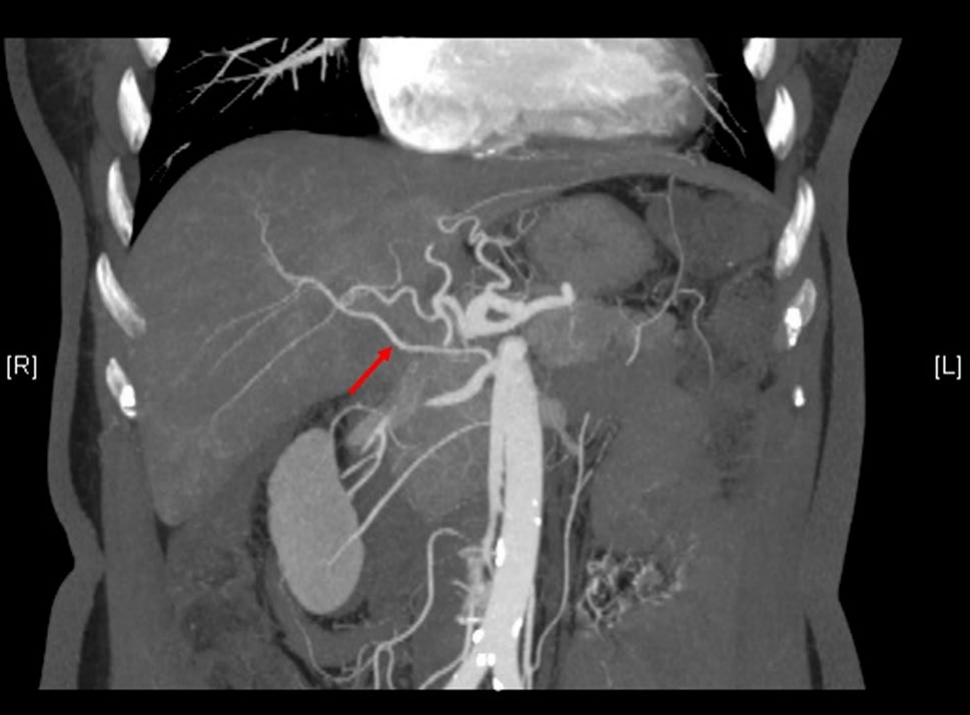
Maximum intensity projection of enhanced CT scan shows that the aRHA, derived from the SMA, ran into the RAL. RPHA was derived from the PHA

**Fig. 2:**
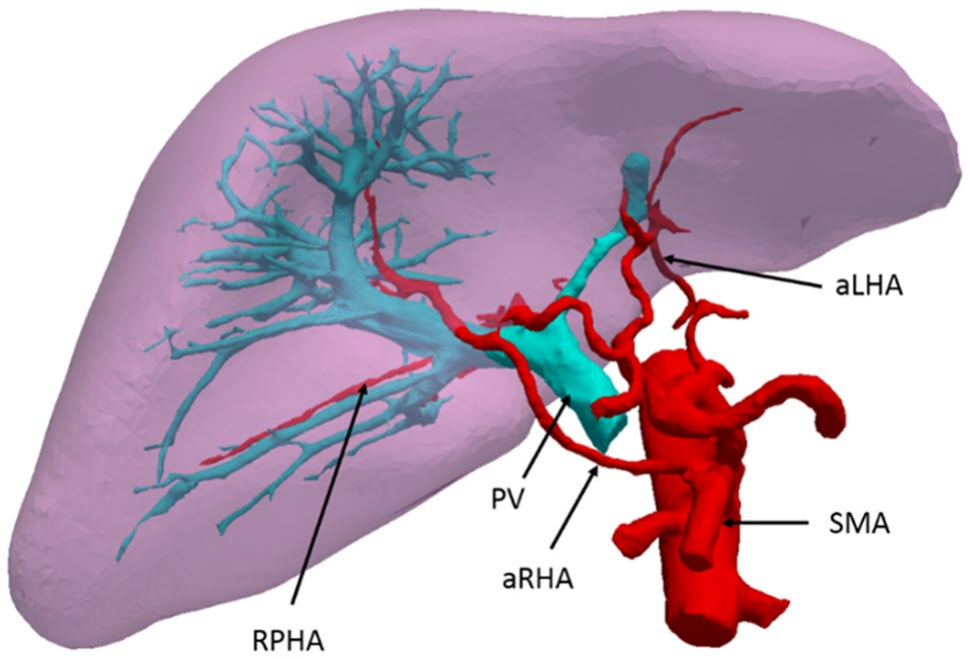
Three-dimensional reconstruction showing the spatial anatomical relationship of the liver, artery, and portal vein. *PV* portal vein, *SMA* superior mesenteric artery, *RPHA* right posterior hepatic artery, *aRHA* accessory right hepatic artery, *aLHA* accessory left hepatic artery

A 67-year-old woman was admitted to our hospital, because she was suffering from upper abdominal discomfort. She was diagnosed as having intrahepatic cholangiocarcinoma. From an enhanced multidetector-row computer tomography (CT) examination, a variation of the hepatic artery was detected (Fig. 1). Using three-dimensional reconstruction software (Hisense computer-assisted surgery system, Qingdao, China), it was demonstrated that an aRHA arose from the SMA, ran through the right posterior side of the portal vein (PV), wrapped round from the back to the front of the right branch of the PV, and had distributed into the right anterior liver (RAL) to provide the blood supplies of segment V and VIII. The patient’s accessory left hepatic artery (aLHA) was shown to originate from the left gastric artery (Fig. 2).

## Discussion

According to Tandler, during embryonic development four visceral branches of the aorta with a ventral longitudinal anastomosis are developing, from which normally the second and third roots and the anastomosis disappear. The first root becomes the coeliac trunk with the left gastric artery, splenic artery, and common hepatica artery branches, while the fourth root becomes the superior mesenteric artery. However, in case of sustained anastomosis and differential branch reductions, different ramifications are occurring [9]. In the present case, an aRHA derived from the SMA and an aLHA from the left gastric artery. According to Michel’s classification, this case should be classified as type VII, and according to a recent review of the literature including 19,013 hepatic artery variants, the combination of aRHAs and aLHAs is the rarest form, occurring in only 0.2% of cases, but an aRHA (type VI) was found in 1.6% of cases [8]. The territory of an RHA coming from SMA is variable from the lateral right sector to the whole liver. However, in the present case, the territory of the aRHA coming from SMA is more medial than the territory of the RHA coming from the PHA.

As a common problem of aRHAs arising from SMA, an aRHA is likely to be injured during pancreaticoduodenectomy. Liver transplantation graft vessel injury must be avoided during back-table procedures. Longer lengths of right posterior hepatic arteries (RPHAs) may be retained if they originate from the SMA, but the artery studied in the present case was obviously shorter in its presented variation. Careful attention must be paid to avoid damage of the right anterior hepatic artery (RAHA) [2, 5, 7]. To identify the blood supply of a tumor and improve the prognosis, separate angiographies of SMA and hepatic arteries during Transhepatic Arterial Chemotherapy and Embolization (TACE) treatment of right hepatic carcinoma is obligatory [6].

Without a crossing variation, an aRHA is located at the right side of the PV and goes into the RPL. Apparently, since it is relatively distant from the hepatic hila and from the confluence of the right and left bile ducts, such a variation has certain advantages in hilar cholangiocarcinoma surgery. However, the present crossed pattern variation is more easily invaded by tumors, because the RAHA and RPHA are both close to the hepatic hila. An RPHA reconstruction may become necessary during an operation [4]. With similar diameter, the SMA-derived RAHA can be used to connected with the stump of the RPHA if hepatic artery reconstruction has to be carried out during surgery. In addition, in this mode of variation, it is not necessary to dissect the RHA behind the bile duct during right anterior lobectomy, thus lowering the technical difficulty of the operation.

Identification and proper management of hepatic artery variations is critical in surgical procedures. Enhanced CT scans and arteriography are important methods to diagnose vascular variations. Three-dimensional reconstruction is useful to show the spatial anatomical structure of vascular variations and facilitates surgical planning [1].
